# The body before the dance somatic codification, elemental self-regulation, and contemplative recognition in the Naṭarāja tradition

**DOI:** 10.3389/fpsyg.2026.1872500

**Published:** 2026-08-05

**Authors:** Swapan Samanta

**Affiliations:** Indoriv Clinical Research Centre, Kolkata, India

**Keywords:** anthropology of emotion, co-constitution, contemplative science, dance cognition, embodied cognition, interoception, Naṭarāja, Nāṭyaśāstra

## Abstract

This article introduces the Somatic Codification Hypothesis (SCH), a theoretical framework that models the *karaṇa* system of the *Nāṭyaśāstra*—the foundational Sanskrit treatise on performance—as a systematic codification of pre-reflective, stimulus-driven bodily responses, alongside, rather than in place of, its established expressive, devotional, and aesthetic readings. The claim advanced here is epistemic and functional, not ontological: I do not argue that the body generates culture, nor that historical practitioners worked outward from physiology to notation. Rather, I argue that treating the tradition *as if* it codifies regulatory events yields a structured and testable research programme that current expressive and computational models do not provide. In addition, three interlocking proposals are developed. First, each codified movement unit (*karaṇa*) is modelled as a repeatable and teachable form of an involuntary regulatory response to a class of stimulus—what I call a “somatic fossil.” This term is used metaphorically for a preserved pattern and not as a claim that the body precedes its cultural shaping. Second, the *Pañcabhūta* (five-element) framework is reinterpreted as a pre-modern phenomenological taxonomy of five interoceptive channels: structural–proprioceptive (*Pṛthvī*), circulatory–fluid (*Jala*), metabolic–thermogenic (*Agni*), respiratory–neural (*Vāyu*), and witness-awareness (*Ākāśa*). Third, the *Naṭarāja* iconography is read as a somatic self-model in which the contemplative declaration *Śivoham* (“I am Śiva”) can be understood as a recognition of the witness-function of awareness. A geometric model is introduced—the Dance Orientation Vector (DOV), the Śiva-Distance metric, ΔŚ = √[Σα (1 − ρα)^2^], and the PDI, PDI = (1/T)∫₀ᵀ ΔŚ(t) dt—which places any performance on a continuous scale between interoceptively led and exteroceptively led engagement. The model relies on measurable physiological, behavioural, and self-report proxies rather than any direct quantification of felt sensation, without ranking the two modes ontologically. The framework is positioned as an embodied extension of predictive processing and is situated explicitly within the contemporary consensus that somatic experience is co-constituted, being simultaneously physiological and cultural. The geometric apparatus is the framework’s central formal lens: It quantifies measurable proxies and the orientation computed from them—rather than felt sensation—and provides a multidimensional coordinate system in which heterogeneous forms of evidence (physiological, behavioural, self-report, and, complementarily, micro-phenomenological and ethnographic) can be related, compared, and turned into falsifiable predictions. Implications are developed primarily for dance movement therapy and further for contemplative science and embodied cognition research.

## 1 Introduction: a complementary view from the body

*Central falsifiable claim*. The Somatic Codification Hypothesis (SCH) predicts that classical Indian dance traditions with distinct elemental emphases will show measurably different interoceptive accuracy profiles (expected effect size *d* > 0.5) and that Performance Deviation Index (PDI) values will differ between audience-present and contemplative performance conditions (*d* > 0.6). Failure to observe these differences under controlled conditions would substantially disconfirm the SCH.

Dance cognition research has yielded important insights into the neural correlates of movement perception (Calvo-Merino et al., 2005), the kinesthetic rapport between the performer and observer (Reason and Reynolds, 2010), and the motor-simulation processes that underlie aesthetic judgment (Cross et al., 2006). In parallel, dance movement therapy has accumulated clinical evidence for the efficacy of structured movement across conditions ranging from depression to PTSD (Koch et al., 2019). What the field still lacks is a systematic account of *why* particular movement forms produce particular somatic and psychological effects—a generative grammar connecting bodily form to bodily function.

This article proposes that one such grammar may already be encoded, in unusually explicit form, in the *Nāṭyaśāstra*^1^ of Bharata Muni and in its iconographic synthesis, the *Naṭarāja*. I offer a reading from the somatic side—from the body’s regulatory processes toward cultural form—and I want to be precise from the outset about what kind of reading this is. It is not a replacement for the decorative–mythological interpretations of art historians, the theological readings of devotional practitioners, the cosmic-metaphorical reading (Capra, 1975), or the aesthetic-philosophical reading (Coomaraswamy, 2011). Rather, it runs alongside them and earns its place not by being more fundamental but by being more testable.

My objective is practical as well as theoretical. The framework is meant to do a specific job: to identify how recognisable dance states correspond to distinct profiles of somatic regulation, so that movement can be matched, in a principled way, to clinical and contemplative ends—most concretely, to the selection of movement-based therapy for a given presentation (§8.3). The geometric apparatus introduced in §5 is what makes this job tractable: Clinical matching involves the comparison of multidimensional regulatory profiles against target states, and such comparisons are, formally, questions of distance within a coordinate space. As I argue in §5.4, the geometry is a coordinate system over measurable proxies and first-person reports—it makes no claim that felt sensation is intrinsically quantifiable—but, within that scope, it is not decorative. It is the lens through which the framework’s distinctions are stated, its predictions rendered falsifiable, and its results rendered comparable across cases and studies.

### 1.1 What kind of claim this is

A framework of this scope can be misread in at least three ways, and the differences matter. It could be taken as an *ontological* claim—that bodies generate culture, with aesthetic form as a secondary residue of biological processes. It could be taken as a *historical or developmental* claim—that the authors of the *Nāṭyaśāstra* consciously worked outward from physiology to notation. Alternatively, it could be taken as an *epistemic and functional* claim—that modelling the tradition as a codification of regulatory events generates predictions one can confirm or disconfirm. Only the third is intended here.

The distinction is not cosmetic. When the prose below describes a *karaṇa* as “derived from” a somatic event, or of aesthetic form as the “terminal product” of a sequence, these phrases indicate the direction in which the *model* reasons, not a sequence in nature or a hierarchy of the real. I make no claim that there exists, anywhere, a stratum of pure bodily response uncontaminated by culture and waiting to be read off. As I argue in §1.3, I do not believe such a stratum is available even in principle. The “somatic fossil” is a metaphor for a pattern preserved in repeatable form; it is not a fossil of nature beneath culture.

### 1.2 One relationship, stated once

A theory that wants to be tested must say plainly what relationship it asserts between movement and regulation and then hold to it. Across earlier drafts, the relationship shifted among four distinct claims—that movement is an *outcome* of regulatory states, a *mechanism* for regulating them, a *structured expression* of them, and a *symbolic representation* of them. These are not equivalent, and conflating them makes the central hypothesis harder, not easier, to evaluate.

I therefore commit to a single relationship and use it consistently: The *karaṇa* is the codified rehearsal of a regulatory pattern. The somatic event is the involuntary regulatory response; the *karaṇa* is the cultural codification of that response into a repeatable, teachable form; and performing the *karaṇa* is rehearsing the regulation in the absence of the original stimulus. On this account, a movement form is not merely an outcome of a bodily state, an outward expression of it, or a symbol that points at it. It is a *practice technology*—at once shaped by a regulatory pattern and an instrument for re-entering it. When I write below that a *karaṇa* “is” a somatic event, this is the compressed form of that single claim: it is the codified, rehearsal-based form of that event’s regulatory pattern. As codification is irreducibly cultural, the form is, in the same breath, a bodily pattern and a cultural artefact—a point I develop next.

### 1.3 Situating the SCH: co-constitution, not primacy

The strongest objection a careful reader can raise against a project like this one is that it smuggles in a discredited hierarchy—biology as the real foundation and culture as a symbolic coating laid over it. The objection is well taken, and both the humanities and the biological sciences have long since left that dichotomy behind. The settled position in both the anthropology of emotion and the social neurosciences is one of interrelation and bidirectional emergence: The body is not a raw datum prior to culture, and culture is not a mere ornament on a biological core (Csordas, 1993; Barrett, 2017).

I accept this consensus without reservation, and I want to reformulate the Somatic Codification Hypothesis (SCH) in its own terms rather than against them. Danced movement is not the discharge of an internal cascade, and it is not the execution of an external code. It is the emergence of a phenomenon that is, at once and inseparably, somatic and semiotic, involuntary and learned, and physiological and historical. The regulatory pattern is real and bodily; the codification that makes it repeatable, nameable, and teachable is cultural; and the danced movement is both at the same time. Lutz's (1988) demonstration that there is no unmediated access to “pure” interoceptive data outside a framework of cultural meaning is not a difficulty the SCH must explain away—it is a premise the SCH should build on. Hennion's (2007) account of taste and bodily sensibility as accomplishments, cultivated through practice and attachment rather than read off from a substrate, describes exactly what guru-paramparā training does to a dancer’s interoceptive field. Mauss’s (1973) “techniques of the body” identified a similar point a century ago: even the most apparently natural bodily acts are culturally transmitted.

*On the performative tension*. There is a real tension in describing a putatively pre-reflective bodily layer through a deeply cultural vocabulary—the *Pañcabhūta*, the *Nāṭyaśāstra*’s taxonomy, the *Naṭarāja*. I do not regard this as a contradiction to be concealed but as a structural feature of any phenomenology of the interior. There is no culture-free language for inner states; heartbeat and breath become *experiences* only once a tradition has given them names and salience. I therefore do not claim that the five elements are the true, pre-cultural names of the interoceptive channels. I claim something weaker and testable—that this particular inherited taxonomy happens to carve the interoceptive field at biologically tractable joints, which is why it operationalises well. Whether it does so better than rival vocabularies is itself an empirical question, not a metaphysical postulate.

Reframed this way, the SCH does not assert the primacy of the pre-reflective. It proposes one disciplined way of conceptualising the tradition, takes its place within a field of legitimate alternatives—including approaches grounded in the cultural construction of taste and perception (Hennion, 2007)—and stakes its value on the predictions it generates rather than on any claim to have found the natural ground of the phenomenon.

### 1.4 Theoretical positioning: an embodied extension of predictive processing

The SCH is positioned as (Seth, 2013; Seth and Friston, 2016), within the broader enactive and embodied-cognition tradition (Varela et al., 1991). Whereas predictive processing models interoception as the brain’s inference about its own bodily states, it has so far lacked a structured mapping of the body’s regulatory dimensions. Expression-based models of dance, for their part, treat movement chiefly as the outward projection of affect, leaving the interoceptive and autonomic processes that precede and shape expression largely unaddressed. The SCH proposes to provide what each lacks: a five-dimensional embodiment mapping (the *Pañcabhūta*) and the hypothesis that classical dance training functions as a systematic recalibration of interoceptive prediction. This alignment is offered as a bridge that requires empirical testing, not as an established equivalence.

## 2 The somatic codification hypothesis

### 2.1 The generative sequence and two modes of engagement

The SCH models the sequence underlying a classical dance form as moving, in the order in which the model reasons, from stimulus through pre-cognitive bodily response and elemental disturbance to aesthetic recognition. The aesthetic form—*karaṇa*, *mudrā*, choreographic unit—is, within the model, the terminal node rather than the origin. None of this implies temporal priority in the dancer or a fall from a natural state.

Consider the difference between a dancer who arrives at a *karaṇa* through the regulatory pattern it codifies and a dancer who executes the same visually correct form with attention turned outward, toward the audience and the geometry of the pose. Earlier drafts described the second case as a “phenomenological inversion” and, at points, as a “loss.” That language is normative, and I withdraw it. These are better understood as two distinct *modes of engagement* with the same movement—one interoceptively led and the other exteroceptively led—each mobilising a different arrangement of body, attention, environment, and intention. Neither enjoys a privileged ontological status. The geometric model in §5 measures the difference between them; it does not adjudicate which is truer, more authentic, or more complete. An audience-oriented performance is not a degraded contemplative one; it is a different thing, with its own coherence and its own value.

By *pre-cognitive bodily response,* I mean processes temporally prior to reflective cognition but not necessarily sub-neural—the rapid regulatory adjustments (postural shifts, breath disruption, and muscular co-contraction) while remaining open to phenomenological attention (Sheets-Johnstone, 2011).

The SCH draws on three contemporary frameworks while extending each. Damasio’s (1994, 2010) somatic marker hypothesis grounds emotional processing in bodily states; the SCH notes that the *Nāṭyaśāstra*’s *rasa*–*bhāva* distinction anticipates the emotion–feeling dichotomy with some precision. Craig’s (2002, 2009) interoceptive model establishes the insular cortex’s role in constructing bodily self-representation; the SCH proposes that the *Pañcabhūta* can be modelled as a pre-modern taxonomy of these channels. Seth and Friston’s (2016) active interoceptive inference account treats interoception as predictive self-modelling; the SCH proposes that classical dance training is a systematic recalibration of those predictions under elemental disturbance.

### 2.2 The Pañcabhūta as a phenomenological taxonomy

The five-element framework—the *Pañcabhūta*^2^—runs through Indian philosophical, medical, and aesthetic thought. The SCH offers a functional reinterpretation, not as metaphysical substances but as phenomenological categories of interoceptive experience, each corresponding to a distinct physiological channel. The mapping is heuristic, claims no historical intent on the part of ancient authors, and—consistent with §1.3—is offered as one culturally given vocabulary that operationalises well, not as the natural names of the channels (see Table 1).

**Table 1:** The Pañcabhūta: from metaphysical substance to interoceptive channel.

Element	Traditional reading	Phenomenological reinterpretation	Physiological correlate	Interoceptive channel
*Pṛthvī*	Earth; solidity	Structural stability; resistance to deformation under force	Skeletal system, postural muscles	Proprioception; gravitational orientation
*Jala*	Water; fluidity	Circulatory continuity; rhythmic self-maintenance	Blood, lymph, and interstitial fluid	Cardiac interoception; fluid-balance sensing
*Agni*	Fire; transformation	Metabolic heat; energy release and thermogenesis	Glucose metabolism and thermogenesis	Thermoreception; metabolic sensing
*Vāyu*	Air; movement	Respiratory–neural sensitivity; breath, tremor, and nerve conduction	Respiratory system andautonomic nervous system	Respiratory interoception; chemoreception
*Ākāśa*	Ether; void	Witness-awareness; the reflexive capacity that observes bodily states	Default mode network, anterior insula	Meta-interoception (awareness of awareness)

Physiological correlates are offered as plausible anchors for future empirical operationalisation, not as established equivalence.

## 3 The Karaṇa as a codified rehearsal: a heuristic decoding

The *Nāṭyaśāstra* codifies 108 *karaṇas*,^3^ fundamental movement units. Subrahmanyam’s (1979, 2003) reconstructions recovered the physical forms of many of these from the temple sculptures at Chidambaram. The present analysis extends that research by proposing origin-functions for a selected set of *karaṇas* within the SCH: the regulatory patterns of which, on this reading, the movements are the codified rehearsal.

These correspondences are heuristic, grounded in movement morphology and inferred physiological function rather than in historical or textual verification. A total of two criteria constrain them: Biomechanical plausibility—the proposed somatic origin must be mechanically consistent with the visible form—and autonomic plausibility—consistent with known autonomic physiology (Porges, 2011). They are not reverse inferences from a movement to a single cause but probabilistic correspondences of the form Stimulus → P(Element | context) (see Table 2).

**Table 2:** Karaṇa decoding: visible form and proposed somatic origin.

Karaṇa	Visible form	Somatic origin (SCH reading)	Element	Stimulus class
*Āviddha*	Body contracts then darts	Startle-recoil; pre-cognitive withdrawal from sudden threat	*Vāyu*	Sudden threat, alarm, unexpected stimulus
*Ūrdhvajānu*	Raised knee, alert posture	Adrenaline-readiness; the body prepares for action before cognitive decision	*Vāyu–Agni*	Perceived danger requiring immediate motor readiness
*Svastika*	Limbs cross; centre protected	Self-containment; the body protects the vital centre under pressure	*Pṛthvī*	External pressure; need for structural consolidation
*Bhramarī*	Spinning, turning	Metabolic heat-spiral; energy seeks discharge without destruction	*Agni*	Psychic intensity; accumulated thermogenic energy
*Recita*	Wrist/limb quiver	Anticipatory tremor; micro-instability under uncertainty	*Vāyu*	Anticipation; unresolved expectation; and nervous preparedness
*Valita*	Neck and trunk curve	Longing-bend; the body curves toward an absent or remembered object	*Jala*	Attraction; recollection; and emotional absence
*Līna*	Torso softens inward	Grief-collapse; structural dissolution as inner space empties	*Ākāśa–Jala*	Loss; surrender; and irreversible separation
*Nikuttaka*	Rapid stamping	Urgency-grounding; structural presence asserted against dissolution	*Pṛthvī–Agni*	Urgent alert and need for decisive action
*Tālapuṣpapuṭa*	Hands converge, offering gesture	Inner gathering; the dispersed organism collects toward the centre before contemplation	*Ākāśa–Vāyu*	Reverence and pre-contemplative settling

Karaṇa examples are heuristic correspondences based on movement morphology and inferred physiological function, constrained by biomechanical and autonomic plausibility. They are not historically verified one-to-one mappings. The table illustrates the compositional logic of the SCH and is not a closed system.

The decisive point is compositional. The *karaṇa* preserves a regulatory pattern in a repeatable form, so that the dancer can rehearse the body’s response to a class of stimulus without the stimulus itself—which is precisely what makes it a practice technology rather than an expression. The dancer training the *Āviddha* is not “expressing” fear; she is rehearsing the *Vāyu* system’s capacity to maintain coherence under the kind of disturbance that fear can be modelled as producing. In addition, because this rehearsal is transmitted through a named and taught form, it is at every step as much a cultural act as a bodily one.

## 4 Rasa as interoceptive recognition

Bharata’s *rasa-sūtra*^4^ holds that *rasa* arises from the conjunction of *vibhāva* (stimulus), *anubhāva* (response), and *vyabhicāri-bhāva* (transitory states). The standard interpretation reads this as a theory of emotional communication between the performer and spectator. The SCH offers a complementary reading: *rasa* can be modelled as the organism’s recognition of its own elemental reorganisation under stimulus—a recognition that is, again, both a bodily event and a culturally schooled one, since what counts as a recognisable pattern is shaped by training (Lutz, 1988; Barrett, 2017).

This aligns with interoceptive inference accounts. Seth (2013) proposes that emotional experience is constituted by the brain’s predictive model of its own interoceptive states. Within the SCH, *rasa* is the aesthetic name for that predictive recognition—the moment the body’s elemental disturbance becomes legible to the awareness that observes it. The correspondence is a theoretical bridge, not a claim of historical equivalence; *rasa* categories function here as labels for modes of interoceptive recognition (see Table 3).

**Table 3:** The Nine Rasas as interoceptive–elemental recognition states (SCH framework).

Rasa	Standard translation	SCH reinterpretation	Elemental profile	Predicted interoceptive signature
*Bhayānaka*	Terror/Fear	Recognition of *Vāyu* crisis: breath fracture and neural mobilisation before cognition	*Vāyu* dominant and *Pṛthvī* destabilised	Predicted respiratory irregularity; elevated HR; sympathetic surge; and proprioceptive instability
*Raudra*	Fury/Rage	Recognition of *Agni* surge: metabolic heat exceeding containment capacity	*Agni* dominant	Predicted thermal rise; muscular tension; and peripheral vasoconstriction
*Karuṇa*	Compassion/Grief	Recognition of *Ākāśa* opening through *Jala* loss: inner space becomes visible as the form loosens	*Ākāśa–Jala*	Predicted respiratory hollowing; lacrimation; cardiac deceleration; and chest-emptying sensation
*Śṛṅgāra*	Desire/Love	Recognition of *Jala–Agni* expansion: the self bends toward completion beyond itself	*Jala–Agni* balanced	Predicted warmth diffusion; vasodilation; deepened circulation; and muscular softening
*Vīra*	Heroism/Resolve	Recognition of *Pṛthvī* ordering: structural stability reasserted under threat	*Pṛthvī* dominant	Predicted postural stabilisation; respiratory regularisation; and tonus increase
*Adbhuta*	Wonder	Recognition of *Vāyu* opening into *Ākāśa*: familiar prediction models give way to a larger pattern	*Vāyu* → *Ākāśa*	Predicted breath suspension; sensory widening; pupil dilation; and cognitive pause
*Hāsya*	Laughter/Comedy	Recognition of *Vāyu* liberation: diaphragm releases and rigidity collapses	*Vāyu* liberated	Predicted diaphragmatic spasm; respiratory pulsing; and parasympathetic activation
*Bībhatsa*	Disgust/Aversion	Recognition of *Agni–Vāyu* defensive purge: the body rejects what cannot be assimilated	*Agni–Vāyu* defensive	Predicted nausea response; recoil; expulsive respiratory pattern; and gastric contraction
*Śānta*	Peace/Tranquillity	Recognition of elemental equilibrium: all systems functioning without corrective override	*Ākāśa* clarified; all balanced	Predicted HRV coherence; respiratory regularity; low sympathetic tone; and maximal interoceptive accuracy

Interoceptive signatures are directional predictions, not empirically verified profiles. They represent the SCH’s testable hypotheses, not established physiological facts. HRV, heart rate variability; HR, heart rate.

Within the SCH, then, *rasa* is not an emotion that the performer projects and the audience receives. It denotes the moment the organism recognises the pattern of its own disturbance and recovery. An audience may participate through a parallel form of interoceptive resonance, a partial replication of the pattern via mirror-system engagement (Calvo-Merino et al., 2005) and autonomic entrainment (Müller and Lindenberger, 2011). This resonance is one of the SCH’s testable predictions, and it is worth noting that it already grants the audience-oriented mode a genuine, non-derivative function.

## 5 A geometric model of dance orientation and elemental deviation

*Scope of the formal core*. Sections 5 and 8 constitute the falsifiable core of the article and stand on their own. The interpretive applications in §6 and §7, as well as the synthesis table in Appendix A, are explicitly *not* load-bearing for the model’s validity; they are generative extensions, and a reader who rejects them can still accept or test the formal model.

The preceding sections argue that a classical dance movement can be modelled as having an interoceptively led orientation, from which it may rotate toward an exteroceptively led orientation. This section translates that argument into a quantitative schema intended as proto-operational guidance for empirical work. The variables I(t) and E(t) are theoretical constructs; §8 specifies measurement proxies. Treating I(t) and E(t) as orthogonal is a first-order approximation, and coupling between them is a direction for refinement.

### 5.1 The dance orientation vector (DOV)

For any movement at time t, two components are defined. The *interoceptive component* I(t) is the degree to which a movement is driven by and attended to internal somatic processes—breath rhythm, heartbeat, proprioceptive feedback, visceral sensation, and elemental disturbance and response—normalised to [0, 1]. Proxies include breath–movement synchrony indices, HRV coherence, and movement–heartbeat phase locking. The *exteroceptive component* E(t) is the degree to which a movement is directed toward external perception—visual symmetry, audience gaze, competitive display, and form-compliance—also normalised to [0, 1]. Proxies include gaze direction, audience-monitoring behaviour, and divergence from the cardiac phase in movement timing.


$$\begin{array}{l} D(t)=I(t)\cdot \hat{i}+E(t)\cdot \hat{e}\;\\ \theta (t)=\arctan[E(t)/I(t)] \end{array}$$

At θ = 0°, orientation is purely interoceptive; at θ = 90°, it is purely exteroceptive. The open interval 0° < θ < 90° captures the variability of real performance. The angle records where on this continuum a given moment sits; consistent with §2.1, it does not rank the endpoints.

### 5.2 The five-element pentagonal model

A single angle is insufficient because the SCH posits five channels that may be oriented independently. For each element *α* ∈ {*Pṛthvī, Jala, Agni, Vāyu, Ākāśa*}, the Elemental Interiority Index is defined as *ρ*α(t) = Iα(t)/[Iα(t) + Eα(t) + *ε*], where ε is a small noise/measurement-error term and ρα ∈ [0, 1]; ρα → 1 marks interoceptive engagement of that channel, while ρα → 0 indicates exteroceptive projection. The five ρ-values define a point on a pentagonal radar plot, with one vertex per channel (see Table 4).

**Table 4:** Pentagonal model: vertex definitions and measurement operationalisation.

Vertex (element)	Interoceptive engagement (ρ → 1)	Exteroceptive projection (*ρ* → 0)	Measurement instruments
*Pṛthvī*	Movement driven by felt gravitational orientation and proprioceptive feedback	Postures executed for visual geometry without proprioceptive attending	Force plates (centre of pressure (COP) variability); postural sway analysis; and EMG of postural muscles
*Jala*	Movement entrained to cardiovascular and fluid-system rhythms	Timing driven by external musical structure regardless of the cardiac phase	HRV analysis; pulse-wave coherence; and movement–heartbeat phase locking
*Agni*	Metabolic heat regulated through movement; intensity follows thermogenic need	Intensity driven by choreographic demand irrespective of metabolic state	Infrared thermography; respiratory gas analysis; and skin conductance
*Vāyu*	Breath leads movement; respiratory patterns shape kinetic phrasing	Breath subordinated to the visual form; held for the aesthetic effect	Pneumography; RSA; and breath–movement synchrony indices
*Ākāśa*	Meta-awareness present: the performer witnesses own somatic processes without identification	Self-referential performance monitoring; concern centred on how one appears	fMRI (DMN activation); EEG frontal-midline theta; MAIA-2; and micro-phenomenological interview

RSA, respiratory sinus arrhythmia; HRV, heart rate variability; DMN, default mode network; COP, centre of pressure; MAIA-2, Multidimensional Assessment of Interoceptive Awareness, Version 2. A minimal viable measurement set: RSA (Vāyu), HRV spectral analysis (Jala), infrared thermography (Agni), force plate COP (Pṛthvī), and MAIA-2 with frontal theta EEG (Ākāśa).

### 5.3 The Śiva-distance (ΔŚ) and performance deviation index (PDI)

The reference state I label the “Naṭarāja ideal” is the limit at which all five channels reach maximum interiority, ρ_ideal = (1, 1, 1, 1, 1). I use the word *ideal* in the technical sense of a limit that anchors a coordinate system, not in the evaluative sense of a state every performance ought to reach. An exteroceptively led performance is not a failed attempt at this limit but a point measured against it. The Śiva-Distance is the Euclidean distance from any actual state to that limit. It is chosen because it provides a geometrically interpretable and dimensionally consistent measure across a five-dimensional normalised space, in which each axis is bounded [0, 1] on an equivalent scale:


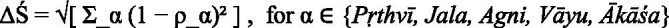


ΔŚ = 0 marks all channels at maximum interiority; ΔŚ = √5 ≈ 2.236 marks complete exteroceptive projection. The Performance Deviation Index (PDI) integrates ΔŚ over a performance of duration T, capturing both mean and temporal dynamics:


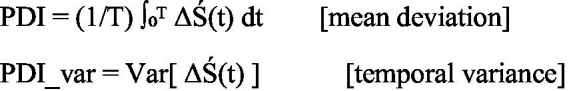

*In plain terms*. These three quantities ask three questions with numbers. *θ* (theta) asks of a single moment: Is this movement listening inward to the body or projecting outward to be seen? ΔŚ (the Śiva-Distance) asks of the whole body at once: Across all five channels together, how far is the dancer from fully listening inward, where 0 means fully inward and approximately 2.24 means fully outward? PDI then averages that distance over an entire performance, and PDI_var asks whether the orientation held steady or kept shifting from moment to moment.

Furthermore, two performances with identical PDI values can differ markedly in PDI_var—one may exhibit consistently low ΔŚ performance, while the other oscillates between periods of high and low ΔŚ. To illustrate, consider two performers executing the same *karaṇa* sequence. Performer A, from an interoceptively led training lineage, shows high breath–movement synchrony (high ρ_*Vāyu*), cardiac phase-locked timing (high ρ_*Jala*), and minimal audience monitoring. Performer B comes from a competition-oriented background and shows low breath–movement synchrony, timing driven by musical structure rather than the cardiac phase, and frequent glances toward adjudicators. The model predicts a PDI of < 0.5 for A and a PDI of > 1.2 for B—a gap that is translatable into measurable differences in HRV, respiratory coherence, and skin conductance. The model describes this gap; it does not declare B’s dancing inferior.

A concrete calculation makes these numbers tangible. For the Dance Orientation Vector (DOV), a single moment with I = 0.8 and E = 0.3 yields *θ* = arctan(0.3/0.8) ≈ 21°—firmly toward the interoceptive pole. For the Śiva-Distance metric, suppose Performer A registers channel scores *ρ* = (0.9, 0.8, 0.7, 0.9, 0.85) for *Pṛthvī, Jala, Agni, Vāyu, and Ākāśa*. Then, for Performer A, ΔŚ = √[(0.1)^2^ + (0.2)^2^ + (0.3)^2^ + (0.1)^2^ + (0.15)^2^] = √0.1725 ≈ 0.42, placing the value close to the somatic limit of 0. For Performer B, scoring ρ = (0.6, 0.3, 0.7, 0.4, 0.2) yields ΔŚ = √[(0.4)^2^ + (0.7)^2^ + (0.3)^2^ + (0.6)^2^ + (0.8)^2^] = √1.74 ≈ 1.32, well toward the exteroceptive end. The metric thus compresses a qualitative difference in how two dancers inhabit the same choreography into a single comparable number, while, as stressed above, it does not rank either as the better dance (see Table 5).

**Table 5:** Predicted PDI profiles for performer types.

Performer type	Predicted PDI	Predicted θ	Pentagon shape	Dominant ρ pattern
Expert: interoceptively led/guru-paramparā lineage	Low (< 0.5)	< 25°	Full, regular pentagon	All ρ ≥ 0.7
Expert: technical/competition emphasis	Moderate (0.5–1.2)	30°–55°	Irregular; *Ākāśa* collapsed	High *Pṛthvī, Agni*; low *Ākāśa*
Competitive dancer (audience-primary)	High (1.2–1.8)	55°–75°	Collapsed; *Jala* and *Ākāśa* near zero	*Pṛthvī* and *Agni* only
Contemplative mover (meditative)	Very low (< 0.3)	< 15°	Full; *Ākāśa* dominant	*Ākāśa* and *Vāyu* highest

PDI values are theoretical predictions requiring empirical validation. Performer types are idealised categories, not discrete groups; the categories index different modes of engagement rather than a ranking of skill. Expected within-category variance is an agenda for initial studies.

### 5.4 What the model quantifies and what it does not

A reasonable objection to any formalism of this kind is that sensation cannot be quantified—that the felt, inward texture of a dance state is fluid, person-dependent, and shaped by more variables than a coordinate can hold. I agree, and I want to state plainly that the SCH does not dispute it. The point is easy to lose in the notation, so it deserves its own section: the model does not take the felt quality of sensation as its measured object. What it quantifies are *proxies* and *orientation*, not qualia.

Every quantity in §5 is computed from independently validated, uncontroversially measurable signals, including physiological proxies (heart rate variability, respiratory sinus arrhythmia, infrared thermography, and centre-of-pressure variability), behavioural proxies (gaze direction and audience monitoring), and a validated interoceptive self-report instrument (the Multidimensional Assessment of Interoceptive Awareness, Version 2, MAIA-2). The Elemental Interiority Index ρα is a ratio computed from these proxies; the Śiva-Distance ΔŚ is therefore a distance between *measured orientation profiles*, not between sensations. The word “distance” is literal with respect to the proxy vector and only metaphorical with respect to anything felt. When the model locates a performance near the interoceptive pole, it is making a claim about breath–movement synchrony, cardiac phase locking, and attentional direction—not a claim to assign a numerical value to what the dancer feels.

The model does not assume that sensation is stable. It is built around the opposite assumption. ΔŚ is an explicit function of time; PDI integrates it over an entire performance precisely because orientation drifts from moment to moment; PDI_var measures how much it wanders; the *ε* term in ρα absorbs measurement noise; and §8 treats person and context dependence—training lineage, age of onset, training modality, and the presence or absence of an audience—not as error to be suppressed but as substantive variables the framework expects to matter. The geometry is a way of tracking change, not of freezing it.

The felt, first-person dimension that genuinely resists quantification is not left out; it is assigned to the methods best suited to it. Throughout the framework, micro-phenomenological interview (Petitmengin, 2006), ethnographic description, and neuroimaging stand alongside the quantitative proxies as co-primary sources of evidence, not as subordinate illustrations of them. The lived texture of a dance state and its physiological signature are different kinds of evidence, and neither substitutes for the other. These methods and the geometry are not in competition; they work at different levels. Ethnography and micro-phenomenology characterise what the channels feel like and how practitioners attend to them; the geometry is what then lets those characterisations be placed in relation to one another, compared across persons and conditions, and turned into the distance-and-deviation predictions of §8. The reason the representation is geometric rather than merely verbal is that the object it represents is irreducibly multidimensional—five channels varying independently and at once—and because the clinical question at the centre of the article, namely, how far a given regulatory profile lies from a target therapeutic state and along which channels, is one that only a metric space can pose precisely. A purely narrative model can describe a single case vividly, and that is exactly the work assigned to the phenomenological strand; it cannot, on its own, render profiles comparable across cases, generate falsifiable effect size predictions, or provide a common language across studies—which is what a therapeutic programme requires and what this formalism is built to provide. The geometry is, in this sense, the connective tissue of the wider research programme of which this article is one part.

## 6 The Naṭarāja as a somatic self-model

*Interpretive boundary*. This section provides an interpretive extension of the SCH to *Naṭarāja* iconography. It is not required for the validity of the formal model (§§5, 8 stand independently) and does not replace existing scholarly interpretations. The reading is functional and offered as one interpretation among several.

The *Naṭarāja*^5^ of Śiva is among the most recognisable images in world art. Coomaraswamy’s (2011) influential interpretation reads each element as a cosmic symbol: the drum as the sound of creation, the fire as dissolution, the raised foot as liberation, the crushed *Apasmāra* as ignorance overcome, the *abhaya mudrā* as divine protection. The SCH offers a complementary reading grounded in the body. The two interpretations are not mutually exclusive, and nothing in the somatic reading requires the cosmic one to be displaced (see Table 6).

**Table 6:** Naṭarāja iconography: cosmic and somatic readings (SCH framework).

Iconographic element	Cosmic reading (Coomaraswamy)	Somatic reading (SCH)	Elemental correspondence
Fire (right hand)	Cosmic dissolution; destruction at the end of the cycle	Metabolic heat—ceaseless thermogenesis through glucose combustion in muscle tissue	*Agni*—the metabolic fire sustaining life through controlled burning
Drum/Ḍamaru (left hand)	Primordial sound of creation	Cardiac rhythm—the first and last involuntary percussion of the living body	*Jala*—tidal pulse of blood; the body’s internal ocean
Gaṅgā (in hair)	Sacred river descending from heaven	Circulatory memory — salt in blood, tears, and sweat recalling the body’s oceanic origin	*Jala*—fluid-system continuity; evolutionary tidal memory
Raised foot	Liberation (mokṣa); transcendence of gravity	Suspension between inhale and exhale—respiratory liberation from gravitational automaticity	*Vāyu*—breath’s capacity to momentarily free the organism from the habitual pattern
Crushed Apasmāra	Ignorance (avidyā) overcome by wisdom	Unconscious automaticity—habitual patterns below awareness, pressed into the ground by conscious attention	*Pṛthvī*—structural habit overcome by structural awareness
Abhaya mudrā	“Fear not”—divine protection	The witness-function of awareness—the dimension never itself disturbed by any disturbance	*Ākāśa*—meta-awareness; the space in which all elemental processes occur
Still vertical axis	The cosmic axis mundi	Phenomenological invariant—awareness remains constant, while interoceptive states fluctuate; measurable via meta-awareness scales	The ρ = 1 state across all elements; ΔŚ = 0

The somatic reading is a functional interpretation within the model, not a claim about historical intent or the primacy of this reading over others.

On this reading, the *Naṭarāja* figures the human body with all five elemental systems simultaneously active, recognised, and at interiority *ρ* = 1. The stillness of the axis is not the absence of movement but the presence of meta-awareness (the *Ākāśa* dimension), which makes the other dimensions legible to themselves. I offer this, once more, as a generative picture rather than a load-bearing claim.

## 7 Contemplative recognition and the post-transcendence model

The contemplative declaration *Śivoham*^6^ (“I am Śiva”) is conventionally read as a mystical identity-claim. Within the SCH framework, a more parsimonious phenomenological reading is available: *Śivoham* may be understood as somatic self-recognition—the recognition that the witness-function of awareness (*Ākāśa*) was never itself among the elements and never itself disturbed by the disturbances it observes. This is offered as a phenomenological interpretation within the framework, not a theological or metaphysical claim, and it is one reading among several, chosen for its structural fit within the model and its parallels with contemporary accounts of minimal selfhood and meta-awareness (Gallagher, 2000; Hölzel et al., 2011; Seth, 2013; Zahavi, 2005).

In the geometric model, this recognition corresponds to the state *θ* → 0°, ΔŚ → 0, reached not through effortful achievement but through the cessation of the outward rotation that produces deviation. This connects to my prior formalisation of perpetual self-transcendence (Samanta, 2026), which modelled liberation as recognition rather than attainment; the present framework provides the somatic–aesthetic ground for that formalisation. The relevant empirical anchor is the phenomenological invariant of the still axis, which the SCH proposes is measurable via meta-awareness scales (e.g., the Nondual Awareness Dimensional Assessment) and insula activation patterns.

## 8 Testable predictions, falsifiability, and experimental directions

For the SCH to function as a scientific hypothesis rather than a philosophical schema, it must be falsifiable, so I state the disconfirming conditions before the positive predictions. The validation programme deliberately employs a mixed-methods design: the quantitative proxies below are to be paired with first-person and ethnographic methods (§5.4), since the felt texture of a dance state and its physiological signature constitute different kinds of evidence and neither of which alone settles the question.

### 8.1 Falsifiability conditions

The SCH would be substantially disconfirmed if: (1) no measurable interoceptive variance is found across classical traditions with distinct elemental emphases—for example, Bharatanatyam and Manipuri practitioners showing identical profiles across all five channels (failure criterion: *p* > 0.05 and *d* < 0.2 for all primary channel comparisons); (2) PDI values do not differ between audience-present/competitive and solitary/contemplative framings of the same sequence (failure criterion: *d* < 0.2); and (3) no correlation is observed between a breath–movement synchrony proxy (ρ_*Vāyu*) and therapeutic outcomes in a DMT intervention specifically designed to raise ρ_*Vāyu* in anxiety disorders (failure criterion: r < 0.2).

### 8.2 Differential interoceptive profiles across dance forms

If the SCH is productive, long-term practitioners of different classical forms should show measurably different interoceptive accuracy profiles, corresponding to the elemental channels their traditions most systematically train. The proposed design is as follows: minimum n = 40 per tradition (power calculation: *d* = 0.5, *α* = 0.05, power = 0.80 yields n ≈ 34 per group; n = 40 allows for attrition), with a minimum of 5 years of practice, compared against age-, sex-, and BMI-matched non-dancer controls. Confounders to be measured and covaried include training hours per week, age of onset, and training modality (guru-paramparā vs. institutional). Consistent with §1.3, training modality is treated not as noise but as a substantive cultural variable expected to shape the interoceptive field (see Table 7).

**Table 7:** Predicted interoceptive advantages by dance tradition.

Dance tradition	Primary elemental training	Predicted interoceptive advantage	Proposed measurement
Bharatanatyam	*Pṛthvī* (structural axis, grounded stance)	Enhanced proprioceptive accuracy and superior postural stability under perturbation	Romberg test variants; COP analysis; and joint-position matching
Odissi	*Jala* (fluid curve, tribhaṅgī)	Enhanced cardiac interoceptive accuracy and superior HRV coherence	Heartbeat counting task and HRV spectral analysis
Kathak	*Agni* (percussive fire, rapid spins)	Enhanced thermoreceptive accuracy and superior metabolic regulation under exertion	Thermal discrimination tasks and metabolic efficiency indices
Manipuri	*Vāyu* (atmospheric subtlety, breath-led movement)	Enhanced respiratory interoception and superior breath–movement synchrony	Respiratory perception tasks and breath-counting accuracy
Contemplative practice (non-performative)	*Ākāśa* (witness-function)	Enhanced meta-interoception; superior ability to observe bodily states without reactive identification	MAIA-2; micro-phenomenological interview; and fMRI insula activation

Expected effect sizes: *d* > 0.5 for primary channel comparisons and *d* > 0.3 for secondary channels. MAIA-2, Multidimensional Assessment of Interoceptive Awareness, Version 2; COP = centre of pressure.

### 8.3 PDI validation studies

The PDI can be validated using a within-subject crossover design comparing the same sequence under two framings: audience-present/competitive (predicted PDI > 1.0; *θ* > 45°) and solitary/contemplative (predicted PDI < 0.5; θ < 20°), with physiological measures across all five channels recorded simultaneously. The recommended sample is n ≥ 30 performers across traditions; the within-subject design minimises individual-difference confounds. The expected effect is a significant PDI reduction under the contemplative framing (*d* > 0.6). Reliability is assessed via test–retest ICC for the PDI across two contemplative sessions conducted 1 week apart (target ICC > 0.70). PDI_var will capture the stability of orientation across the performance arc. This comparison is the heart of the empirical programme, and it is also where the co-constitution thesis earns its keep: the manipulation is *contextual and intentional*, not anatomical, which is exactly what one expects if orientation is jointly set by body and situation.

### 8.4 Implications for dance movement therapy

The clinical application is, for this framework, the primary point—the question of which movement helps which presentation and why. The SCH offers a theoretically grounded basis for matching movement interventions to clinical presentations. These are hypotheses, not treatment claims, and require controlled trials before any therapeutic recommendation. A DMT intervention designed to raise ρ_*Vāyu* (breath–movement integration) is predicted to show specific efficacy for anxiety disorders, modelled as *Vāyu*-dysregulation, over and above non-specific movement. An intervention targeting ρ_*Pṛthvī* (structural grounding) is predicted to address dissociative conditions, modelled as proprioceptive disconnection. Together, these conditions generate a matrix of element-specific therapeutic hypotheses testable within existing DMT frameworks (Koch et al., 2019; Van der Kolk, 2014). As the clinical question is finally about lived sensation as much as physiology, these hypotheses are intended to be evaluated using a combination of physiological measurement, validated interoceptive self-report (MAIA-2), micro-phenomenological interview (Petitmengin, 2006), and, where available, neuroimaging, with qualitative and quantitative strands carrying equal weight.

## 9 Conclusion

The Somatic Codification Hypothesis proposes that the *Naṭarāja*–*Nāṭyaśāstra* complex preserves a phenomenological insight of some precision: that dance can be modelled as elemental conditioning, that the body can be modelled as the site of an ongoing five-channel regulatory process as much as a vehicle for expression, and that the recognition called *Śivoham* can be read as the moment in which this somatic process becomes self-transparent. The geometric formalisation—the Dance Orientation Vector, the pentagonal interiority model, and the Śiva-Distance—translates the insight into a form amenable to empirical testing.

The model generates specific, falsifiable predictions about interoceptive profiles across traditions, autonomic regulation under different performance framings, and element-specific mechanisms in movement-based therapy. It positions the SCH as an embodied extension of predictive processing, supplying the structured embodiment mapping that prior accounts have lacked. The Naṭarāja ideal—ΔŚ = 0—is not a mystical endpoint but a formal limit that anchors the coordinate system and gives the predictions direction.

I want to conclude by being clear about what the framework does not claim. It does not claim to have found the natural ground of dance beneath its cultural surface; on the contrary, it works throughout with a culturally given vocabulary and treats interoceptive experience as itself culturally schooled. If the framework is even partly productive, its broader interest lies in a question that must be posed carefully: whether aesthetic traditions function, among their many roles, as repositories of somatic knowledge—embodied phenomenological maps whose experiential dimension can be more or less foregrounded across different modes of practice. Recovering that dimension would not constitute a return to pre-modern metaphysics, nor a retreat into naturalism, but rather a recognition that the body’s regulatory grammar and the cultural forms that shape it have always been entangled. The body was always already moving; tradition is one of the ways it has learned to recognise what it was doing. This article offers the theoretical scaffold; the empirical programme lies ahead.

## Footnotes

1 The Nāṭyaśāstra, attributed to the sage Bharata and conventionally dated between roughly the 2nd century BCE and the 2nd century CE, is the foundational Sanskrit treatise on drama, dance, and music. It systematises performance into codified components, of which the karaṇa is the basic unit of movement.

2 The Pañcabhūta are the five “great elements” of classical Indian thought—Pṛthvī (earth), Jala (water), Agni (fire), Vāyu (air), and Ākāśa (ether/space). The scheme organises matter and experience across Indian philosophy, the medical tradition of Āyurveda, and aesthetics. It is itself a cultural classification, not a neutral description of nature; the present paper treats the elements not as physical substances but as a phenomenological taxonomy of interoceptive channels (Table 1).

3 A karaṇa is one of the 108 codified units of movement enumerated in the Nāṭyaśāstra, each combining a posture, a hand-and-limb gesture, and a transition between them. Their sculpted forms survive on the temple gopurams at Chidambaram and were reconstructed in the modern era by Subrahmanyam (1979, 2003).

4 In Indian aesthetics, rasa (literally “juice” or “flavour”) is the distilled aesthetic emotion evoked in a sensitive spectator, while bhāva denotes the underlying emotional states that give rise to it. The rasa-sūtra is Bharata’s formula describing how rasa arises from the conjunction of stimulus (vibhāva), response (anubhāva), and transitory feeling (vyabhicāri-bhāva). The nine canonical rasas appear in Table 3.

5 Naṭarāja (“Lord of Dance”) is the iconographic form of Śiva performing the cosmic dance (ānanda-tāṇḍava), most familiar from South Indian bronzes: a four-armed figure encircled by a ring of flame, holding a drum (ḍamaru) and fire, one hand raised in the gesture of reassurance (abhaya mudrā), one foot lifted and the other pressing down a dwarf figure (Apasmāra). Coomaraswamy (2011) is the classic interpretive source.

6 Śivoham (“I am Śiva”) is a nondual contemplative declaration associated with Advaita and Śaiva traditions, asserting the identity of individual awareness with the principle named Śiva. The present paper reads it functionally — as a recognition of the witness-function of awareness — rather than as a theological identity-claim.

## References

[ref1] Barrett L. F. (2017). The theory of constructed emotion: an active inference account of interoception and categorization. Soc. Cogn. Affect. Neurosci. 12, 1–23. doi: 10.1093/scan/nsx060, 27798257 PMC5390700

[ref2] Calvo-Merino B. Glaser D. E. Grèzes J. Passingham R. E. Haggard P. (2005). Action observation and acquired motor skills: an fMRI study with expert dancers. Cereb. Cortex 15, 1243–1249. doi: 10.1093/cercor/bhi007, 15616133

[ref3] Capra F. (1975). The Tao of Physics: An Exploration of the Parallels between Modern Physics and Eastern Mysticism. Berkeley, CA, USA: Shambhala Publications.

[ref4] Coomaraswamy A. K. (2011). The Dance of Śiva: Fourteen Indian Essays. Revised Edn. New Delhi, India: Munshiram Manoharlal Original work published 1924.

[ref5] Craig A. D. (2002). How do you feel? Interoception: the sense of the physiological condition of the body. Nat. Rev. Neurosci. 3, 655–666. doi: 10.1038/nrn894, 12154366

[ref6] Craig A. D. (2009). How do you feel — now? The anterior insula and human awareness. Nat. Rev. Neurosci. 10, 59–70. doi: 10.1038/nrn2555, 19096369

[ref7] Cross E. S. Hamilton A. F. d. C. Grafton S. T. (2006). Building a motor simulation de novo: observation of dance by dancers. NeuroImage 31, 1257–1267. doi: 10.1016/j.neuroimage.2006.01.033, 16530429 PMC1821082

[ref8] Csordas T. J. (1993). Somatic modes of attention. Cult. Anthropol. 8, 135–156. doi: 10.1525/can.1993.8.2.02a00010

[ref9] Damasio A. R. (1994). Descartes' Error: Emotion, Reason, and the Human Brain. New York, NY, USA: Putnam.

[ref10] Damasio A. R. (2010). Self Comes to Mind: Constructing the Conscious Brain. New York, NY, USA: Pantheon Books.

[ref11] Gallagher S. (2000). Philosophical conceptions of the self: implications for cognitive science. Trends Cogn. Sci. 4, 14–21. doi: 10.1016/S1364-6613(99)01417-5, 10637618

[ref12] Hennion A. (2007). Those things that hold us together: taste and sociology. Cult. Sociol. 1, 97–114. doi: 10.1177/1749975507073923

[ref13] Hölzel B. K. Lazar S. W. Gard T. Schuman-Olivier Z. Vago D. R. Ott U. (2011). How does mindfulness meditation work? Proposing mechanisms of action from a conceptual and neural perspective. Perspect. Psychol. Sci. 6, 537–559. doi: 10.1177/1745691611419671, 26168376

[ref14] Koch S. C. Riege R. F. F. Tisborn K. Biondo J. Martin L. Beelmann A. (2019). Effects of dance movement therapy and dance on health-related psychological outcomes: a meta-analysis update. Front. Psychol. 10:1806. doi: 10.3389/fpsyg.2019.01806, 31481910 PMC6710484

[ref15] Lutz C. (1988). Unnatural Emotions: Everyday Sentiments on a Micronesian Atoll and their Challenge to Western Theory. Chicago, IL, USA: University of Chicago Press.

[ref16] Mauss M. (1973). Techniques of the body. Econ. Soc. 2, 70–88 (Original work published 1934). doi: 10.1080/03085147300000003

[ref17] Müller V. Lindenberger U. (2011). Cardiac and respiratory patterns synchronize between persons during choir singing. PLoS One 6:e24893. doi: 10.1371/journal.pone.0024893, 21957466 PMC3177845

[ref18] Petitmengin C. (2006). Describing one's subjective experience in the second person: an interview method for the science of consciousness. Phenomenol. Cogn. Sci. 5, 229–269. doi: 10.1007/s11097-006-9022-2, 30311153

[ref19] Porges S. W. (2011). The Polyvagal Theory: Neurophysiological Foundations of Emotions, Attachment, Communication, and Self-Regulation. W. W. Norton. New York, NY, USA.

[ref20] Reason M. Reynolds D. (2010). Kinesthesia, empathy, and related pleasures: an inquiry into audience experiences of watching dance. Dance Res. J. 42, 49–75. doi: 10.1017/s0149767700001030

[ref21] Samanta S. (2026). Liberation mathematics I: the behavioral and consciousness definition of perpetual self-transcendence. Front. Psychol. 17:1741096. doi: 10.3389/fpsyg.2026.1741096, 42157983 PMC13180822

[ref22] Seth A. K. (2013). Interoceptive inference, emotion, and the embodied self. Trends Cogn. Sci. 17, 565–573. doi: 10.1016/j.tics.2013.09.007, 24126130

[ref23] Seth A. K. Friston K. J. (2016). Active interoceptive inference and the emotional brain. Philos. Trans. R. Soc. B 371:20160007. doi: 10.1098/rstb.2016.0007, 28080966 PMC5062097

[ref24] Sheets-Johnstone M. (2011). The Primacy of Movement. Expanded 2nd Edn. Amsterdam, Netherlands: John Benjamins.

[ref25] Subrahmanyam P. (1979). Karaṇas in Indian Sculpture: A Study of the 108 Karaṇas of the Nāṭyaśāstra as Represented in Temple Sculpture. Mumbai, India: Bhulabhai Memorial Institute.

[ref26] Subrahmanyam P. (2003). Karaṇa — Common Dance Codes of India and Indonesia, vol. 1–3. Chennai, India: Nrithyodaya.

[ref27] Van der Kolk B. A. (2014). The Body Keeps the Score: Brain, Mind, and Body in the Healing of Trauma. New York, NY, USA: Viking.

[ref28] Varela F. J. Thompson E. Rosch E. (1991). The Embodied Mind: Cognitive Science and Human Experience. Cambridge, MA, USA: MIT Press.

[ref29] Zahavi D. (2005). Subjectivity and Selfhood: Investigating the First-Person Perspective. MIT Press.

