# Supplementary material for: The body before the dance somatic codification, elemental self-regulation, and contemplative recognition in the Naṭarāja tradition
Source: Front Psychol. 2026 Aug 5;17:1872500. doi: 10.3389/fpsyg.2026.1872500 (PMC13485791; doi:10.3389/fpsyg.2026.1872500)
Supplement: Supplementary file 1 [file Table_1.pdf]

Supplementary Material

Supplementary Table 1 | Heuristic synthesis table (operational schema).

Status of this table. This synthesis is illustrative, not load-bearing. It demonstrates the compositional logic of the framework and is not part of the falsifiable core (§§5, 8 of the main article). Read it from the Stimulus column rightward; it is not a closed system. The final two rows are speculative phenomenological extensions and should be treated as exploratory, not as operational predictions.

Stimulus	Bodily response	Element	Rasa	Karaṇa	Śiva-dance mode	Layer / function
**Sudden threat**	Breath breaks and limbs move before thought	Vāyu → Pṛthvī	Bhayānaka	Āviddha	Kālika	I. Core Crisis — Instinctive Breakdown
**Humiliation**	Chest tightens and heat rises	Agni	Raudra	Nikuttaka	Rudra	I. Core Crisis
**Destabilising question**	Cognitive delay and memory flickers	Vāyu	Adbhuta	Vartita	Sandhyā	II. Awareness Break — Transitional
**Shock absorption**	Knees tremble and spine holds	Pṛthvī	Vīra	Svastika	Śakti axis	III. Elemental Revelation
**Hunger / longing**	Hollow belly and pulse deepens	Jala	Śṛṅgāra	Valita	Gaurī	III. Elemental Revelation
**Silence / surrender**	After tears: awareness remains	Ākāśa	Śānta	Līna	Anugraha	III. Elemental Revelation
**Mastery missed origin**	Grief + clarity simultaneously	Ākāśa opens	Karuṇa	Līna	Saṁhāra	IV. Philosophical Breakthrough
**Solitude; no audience**	Spontaneous movement emerges	All five balanced	Śānta	Tālapuṣpapuṭa	Ānanda	V. Integration — Self-Reconstitution
**“What danced before first movement?”**	Silence and question dissolves	Ākāśa absolute	Śānta	Dissolved	Pre-Tāṇḍava	VI. Transcendent Silence — Speculative †

Rasa categories are functional labels for modes of interoceptive recognition, not claims about historical or textual intent. *Karaṇa* examples are heuristic correspondences, not one-to-one or historically verified mappings.

† Rows marked Speculative represent exploratory phenomenological hypotheses beyond the core testable claims and should not be taken as operational predictions.

Novelty and Scope Statement

Novelty

This paper introduces the Somatic Codification Hypothesis (SCH), a framework that models classical dance forms — specifically the *karaṇa* system of the *Nāṭyaśāstra* — as codifications of pre-reflective, stimulus-driven regulatory patterns, complementing rather than displacing expressive and representational readings. Its primary innovation is to add a disciplined, testable view from the somatic side of dance cognition, framed throughout as a functional and epistemic claim and situated within the contemporary consensus on the co-constitution of somatic experience.

Key contribution 1 — functional reinterpretation of classical aesthetics. Rasa, *karaṇa*, and *Pañcabhūta* are reconceived as components of a phenomenological–interoceptive system, aligning pre-modern aesthetic theory with embodied cognition and predictive processing. The mapping is explicitly heuristic; no claim of historical intent is made.

Key contribution 2 — formal quantitative model. The DOV, the Elemental Interiority indices (ρα), the Śiva-Distance (ΔŚ), and the Performance Deviation Index (PDI, PDI_var) constitute a geometric framework for quantifying where a movement sits between interoceptively-led regulation and exteroceptively-led display, without ranking the two.

Key contribution 3 — testable predictions with explicit falsifiability. The model yields hypotheses with specified effect sizes ($d > 0.5$), sample-size justifications, confounder controls, and failure criteria. Falsifiability is operationally defined in §8.

Scope

The paper operates at the intersection of theoretical psychology, embodied cognition, and contemplative science, proposing a conceptual and formal model rather than an empirical study. *Theoretical scope:* a cross-disciplinary framework integrating classical Indian aesthetic theory with contemporary models of interoception and predictive processing, claiming functional productivity rather than historical intent. *Methodological scope:* the geometric model is a proto-operational schema; measurement strategies and proxies are specified, while full validation is named as future work. The geometry is the framework's formal core and operates over measurable proxies rather than over sensation itself; it gives first-person and ethnographic description equal standing as inputs (§5.4) while supplying the multidimensional coordinate system in which those inputs become comparable and the predictions of §8 become falsifiable. *Empirical scope:* the testable predictions, with effect sizes, sample-size justifications, confounder controls,

and falsifiability thresholds, are offered as the foundation for an experimental programme in dance cognition and movement-based therapy.

The SCH stands in the tradition of theoretical models in cognitive science that precede and structure empirical investigation — analogous in that respect to the predictive processing framework itself before the accumulation of neuroimaging evidence. Its value lies not in resolving empirical questions but in generating them with enough precision to make resolution possible.
